# Virulence characteristics of five new *Campylobacter jejuni* chicken isolates

**DOI:** 10.1186/1757-4749-5-41

**Published:** 2013-12-13

**Authors:** Lavinia Stef, Ada Cean, Aida Vasile, Calin Julean, Dan Drinceanu, Nicolae Corcionivoschi

**Affiliations:** 1 School of Animal Sciences and Biotechnology, Banat University of Agricultural Sciences and Veterinary Medicine – King Michael I of Romania, Calea Aradului nr. 119, Timisoara, Romania; 2 National Children Research Centre, Our Lady’s Hospital for Sick Children, Crumlin Road, Dublin 12, Ireland; 3 Dunarea de Jos University, Galati, Romania

## Abstract

*Campylobacter* enteritis has emerged as one of the most common forms of human diarrheal illness. In this study we have investigated the virulence potential of five new *C. jejuni* chicken isolates (RO14, RO19, RO24, RO29 and RO37) originated from private households in the rural regions of Banat and Transylvania in Romania. Following isolation and *in vitro* virulence assay, on HCT-8 cells, our results show that all the *C. jejuni* chicken isolates overcome the virulence abilities of the highly virulent strain *C. jejuni* 81-176. Motility, an important virulence factor was significantly improved in all the new chicken isolates. The ability to survive to the antimicrobial activity of the human serum, to resist to the violent attack of bile acids and to survive in the presence of synthetic antibiotics was increased in all the chicken isolates. However, these were statistically significant only for isolates RO29 and RO37. In conclusion our study shows, based on invasiveness and motility, and also on the data provided by the serum and bile resistance experiments that all the new chicken isolates are able to infect human cells, *in vitro*, and could potentially represent a health hazard for humans.

## Introduction

*Campylobacter jejuni* is a leading cause of enteric illness in many the western world, developing countries. Within European Union there were 198 000 confirmed cases of campylobacteriosis in 2009 [1]. *Campylobacter jejuni* belongs to the group of thermo-tolerant bacteria being the most frequent cause of gastrointestinal diseases in humans. *Campylobacter* enteritis is considered to be a food-bourne disese rather than food poisoning, with infections being derived from a range of foods and also water-based environmental sources. Asymptomatic infections, watery and bloody diarrhea have been reported in humans. Epidemiological studies have shown that human *Campylobacter* infection may vary according to geographical area and even with age [2].

*Campylobacter jejuni* is present in the intestinal tract of a wide variety of wild and domestic animals. The contamination of the retail products occurs by de-feathering, evisceration and dipping during slaughtering [3]. In chickens, *C. jejuni* colonizes the mucus overlying the epithelial cells primarily in the ceca and the small intestine but may also be recovered from elsewhere in the gut and from the spleen and liver [4]. Although *C. jejuni* is not likely to grow or survive well in foods often between 40-85% of retail poultry carcasess are *C. jejuni* postive [5]. Furthermore recent studies suggest that ingestion of a small number of *C. jejuni* organisms will result in human illness [6]. Little is known about the incidence of *C. jejuni* infections in Estern Europe and in particular in Romania. Once the Eastern European countries joined European Union it became of extreme importance for the food industry to assess and investigate the field situation in regards to the existent *C. jejuni* strains and to study their potential virulence to humans.

The origin of these strains seems to be important for their virulence and it was reported that there is a correlation between pathogenicity and geographic area of origin of *Campylobacter* strains [7]. Several virulence factors are considered to be important for *C. jejuni* induced enteritis, such as adhesion and invasion of epithelial cells [8], motility [9], serum resistance [10] and resistance to bile salts [11,12]. Poultry meat is an important reservoir and source of *C. jejuni* infection in Romania but little is known in regards to the virulence of *C. jejuni* strains currently present within the poultry farms. In this study we have investigated these virulence characteristics by comparing *in vitro*, the five chicken isolates from Banat-Transylvania region of Romania with the *C. jejuni* 81-176 human isolate which today is the model strain of choice for invasiveness and virulence in the literature.

## Material and methods

### Bacterial strains and growth conditions

*C. jejuni* strains (n = 5) were isolated from cloacal swabs of broilers, conventionally housed raised in small family farms within the regions of Banat and Transylvania in Romania, in 2013. These strains have been named RO14, RO19, RO24, RO29 and RO37. *C. jejuni* 81-176 was also used as control strain in the virulence experiments. All strains used in this study were stored at -80°C in Mueller-Hinton broth containing 20% (v/v) glycerol until required. Strains had been minimally passaged *in vitro* before storage and subsequent testing. When required, bacteria were inoculated on Mueller-Hinton agar containing selective Skirrow’s antibiotics (Oxoid) and grown under microaerobic conditions (5% CO_2_, 5% O_2_, 90% N_2_) at 42°C. After 24 h growth, a loopful of bacteria was inoculated into biphasic media containing Mueller-Hinton agar and RPMI 1640 tissue culture media supplemented with 10% fetal calf serum. This was cultured for 18 h at 42°C microaerobically for invasion assays. These conditions were determined in preliminary studies as optimum growth conditions for the invasion assay. The isolation of strains has been done accordingly to the legislation in place (Law 471/2002 and government ordinance 37/2002) under the supervision of National Sanitary Veterinary Agency. The ethics committee of Banat University of Agricultural Sciences and Veterinary Medicine – King Michael I of Romania, approved this work.

### Bacterial isolation and identification

*Biological material*: Chicken broilers from 6 intensive breeding facilities from the West part of Romania. From each breeding unit 3 animals were taken for study, a total of 18 animals were used for this study. *Sample collection*: Cloaca swabs were recovered using a sterile cotton swab, after collection the sample was sealed into a sterile plastic tube and transported to the laboratory. The fecal samples were collected from cecum directly into the laboratory, under sterile conditions, for this the cecum was recovered from the animals into a sterile petri dish and transported in the laboratory. Within 15-20 minutes from collection the samples were processed. *Bacterial isolation*: The sample from cloaca swab was dissolved into 900 μl sterile water, after that serial dilutions were performed until 10^5^ and 100 μl of each dilution was plated on to 9.5 cm Petri dish with Muller Hinton Agar media (CM0337, Thermo scientific), prepared according to the producer instructions. For the feces sample approximately 100 μl of the cecum content was diluted into 900 μl of sterile water, and serial dilutions was performed from this. Dilutions from 10^2^ to 10^6^ were plated on the same media as swab samples. After 72-96 hours of incubation at 37°C, in microaerophilic conditions, colonies with *Campylobacter sp*. aspect were picked and inseminated on Muller-Hinton agar media supplemented with campylobacter selective supplement (Skirrow, SR0069, Thermo scientific). All plates were incubated at 37°C in microaerophilic conditions using CampyGen AGS (CN0025A, Thermo scientific). The identification of the isolates was performed with conventional (sodium hippurate hydrolysis and commercial identification system (Api CAMPY system, bioMerieux, France).

### Infection assays

The gentamicin protection assay was used to test the ability of *C. jejuni* chicken isolates by comparison with the highly virulent strain *C. jejuni* 81-176 to adhere and invade human instestinal epithelial cells [10]. Briefly, HCT-8 cells were grown (60% confluence) for 15 to 18 h in six-well tissue culture plates at a concentration of 1 × 10^5^ cells per well. Plate grown *C. jejuni* 81-176 wild type and *C. jejuni* chicken isolates were washed and re-suspended in tissue culture medium at an OD_600_ of 0.4. The HCT-8 cells were washed with PBS, and 2 ml of fresh culture medium was added to each well. Bacteria were added to give a multiplicity of infection of 10. Tissue culture plates were centrifuged at 250 × g for 5 min and incubated for 3 h at 37°C in 10% CO_2_. To quantify the number of cell-associated bacteria, infected monolayers were washed at least three times with PBS and treated with 0.1% Triton X-100 in PBS at 37°C for 30 minutes. Tenfold dilutions of each well were plated onto the appropriate agar and colonies enumerated after 3 days of incubation. To quantify the number of bacteria that invaded HCT-8 cells, the infected monolayers were washed with PBS and tissue culture medium (2 ml) containing gentamicin (400 μg/ml) was added to half the wells, and medium with no antibiotic was added to the remaining wells. The tissue culture plates were then incubated for a further 2 h at 37°C and washed with PBS. HCT-8 cells were lysed by the addition of 100 μl of 0.1% Triton X-100 in PBS and incubated for 10 to 15 min at 37°C. Tenfold dilutions of the contents of each well were plated on Mueller Hinton agar and colonies were enumerated after 3 days of incubation. Invasion efficiency was calculated as the average of the total number of CFU/total initial inoculum. *C. jejuni* 81-176 passaged in RPMI 1640 (without cells) was also tested for the ability to adhere to and invade HCT-8 cells. The experiments were conducted on three separate occasions. Results for a representative experiment are presented. The error bars represent standard deviations for three separate wells.. The significance of differences in adhesion and invasion between samples was determined using the Student *t* test. A P value of <0.05 was defined as significant.

### Motility assays

The motility experiments were done according with the method previously described [13]. Five microliters of culture was inoculated into the center of the Mueller Hinton agar plates (0.4% agar), and the diameter of the resulting swarms was measured the next day, following incubation for 24 hours at 42°C in microaerbic conditions. Motility was characterized like an area of growth around the central point of the plate and measured in millimeters.

### Serum resistance

The sensitivity of bacteria to human serum (Invitrogen) was measured. *C.jejuni* 81-176 wild type and the chicken isolates (RO14, RO19, RO24, RO29 and RO37) were plate-grown and resuspended in fresh tissue culture medium (RPMI 1640) to give an OD_600_ of 0.1. Five microliters of bacterial suspension was added to duplicate wells of a six-well plate containing 800 μl of Mueller-Hinton broth and 200 μl of active pooled human serum or to separate wells containing 800 μl of Mueller-Hinton broth and 200 μl heat-inactivated human serum. The plates were incubated for 1 h at 37°C under microaerobic conditions. Bacteria from each well were diluted 10-fold and plated on dry Mueller-Hinton agar plates. The plates were incubated at 37°C under microaerobic conditions. Colony counts were performed after an incubation time of 2 days. All assays were conducted in triplicate and repeated independently three times.

### Resistance to bile salts

MH broth was supplemented with 4.0% (wt/vol) bile salts and 50 mL of media was transferred into 100 ml bottles for each treatment for the chicken isolates and *C. jejuni* 81-176. The medium was then autoclaved and, once cooled, 1 ml of either chicken isolates or *C. jejuni* 81-176 cells were transferred to each bottle. This experiment was carried out in triplicate. The bottles were left in optimum growth conditions for 24 hours after which 20 μl was removed and diluted with 100 μl of water. The series of dilutions were then plated as before and a second set of samples were obtained and diluted after a further 18 hours. Viable cell counts for each plate were determined at the end of the incubation period.

### Antimicrobial resistance

Six antibiotics (nalidixic acid, ciprofloxacin, erythromycin, ampicillin, amoxicillin-clavulanic acid and gentamicin) were tested using the disk E test (Solna, Sweeden). The bacterial inoculum was adjusted to 0.5 McFarland standard turbidity and Mueller–Hinton agar supplemented were used. Bacterial suspension was inoculated in Mueller-Hinton agar Petri dishes. When the plates were dry, six antimicrobial disks were applied per plate and incubated over night in microaerobic conditions at 37°C.

#### Statistical analysis

Experiments were conducted on at least three separate occasions in triplicates. Results are presented as the means ± standard deviations (error bars) of three replicate experiments. Graphs were drawn using Prism, and the unpaired Student *t* test was used to estimate statistical significance. A P value of <0.05 was considered significant.

## Results

### Adhesion and invasiveness of *C. jejuni* strains isolated from poultry

**Figure 1:**
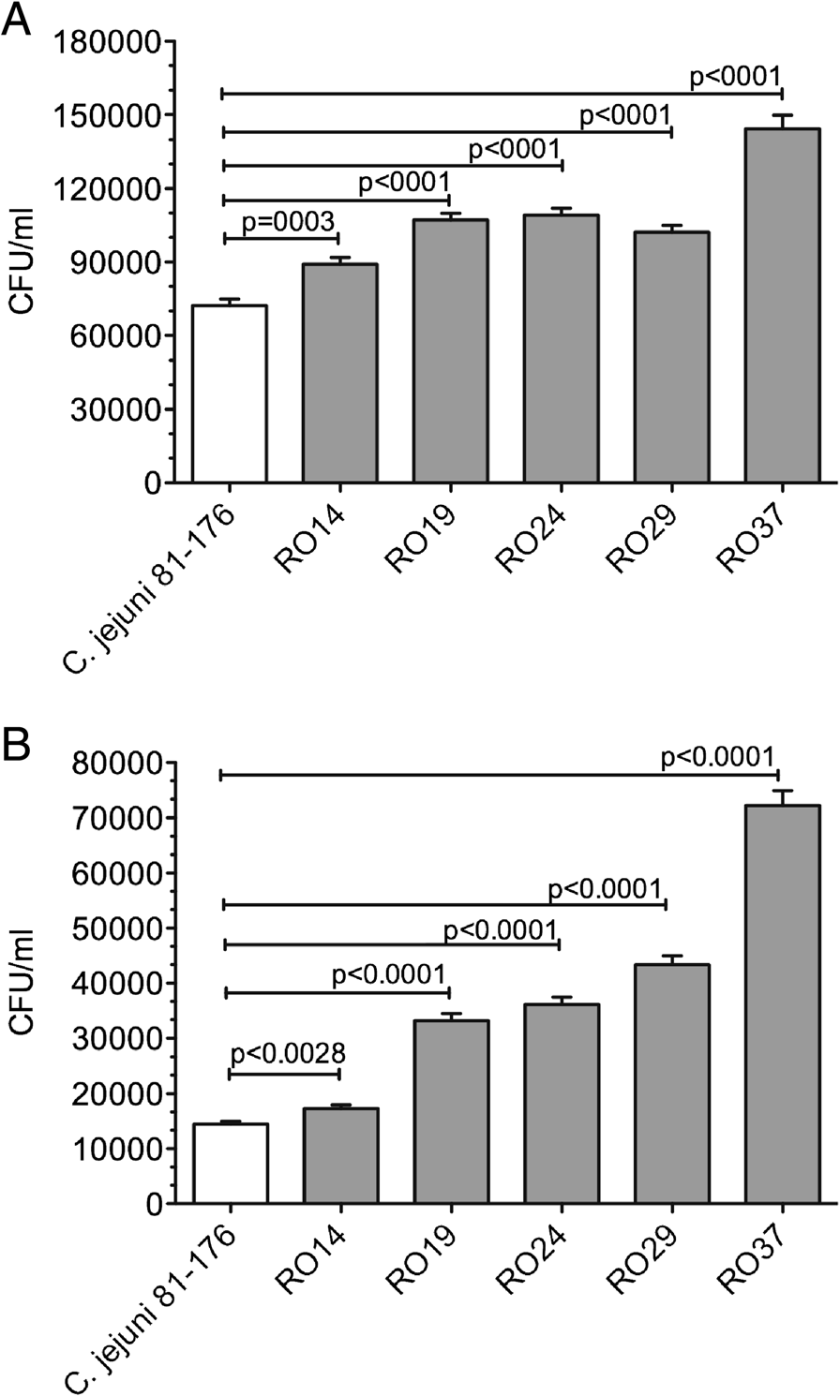
**Increased adhesion and invasion for all chicken isolates. (A)** adhesion to HCT-8 cells, **(B)** invasion of HCT-8 cells of *C. jejuni* 81-176 and *C. jejuni* chicken isolates RO14, RO19, RO24, RO29 and RO37. Statistical significance (Student’s *t* test) relative to the level of *C. jejuni* 81-176 strain is indicated. The experiments were done in triplicate and on three separate occasions. The error bars represent standard deviations for six separate wells.

The virulence of the five chicken isolates (RO14, RO19, RO24, RO29 and RO37) was tested using HCT-8 cells and adhesion/invasion were compared with the model strain *C. jejuni* 81-176, to correct for experimental variation. Invasiveness varied considerably between the investigated strains, as is shown in Figure 1A (adhesion) and B (invasion). Following gentamicin protection assay we have shown that the total adhesion of the chicken isolates is significantly increased compared to the total adhesion of *C. jejuni* 81-176. From the distribution profile obtained the most significant increase in adhesion was recorded for isolate RO37. Normally adhesion levels are not always associated with an increase in the number of bacteria that penetrate the epithelial cells. However, for the *C. jejuni* chicken isolates described in this study this was not the case as higher levels of internalization were found in all isolated strains. Similarly, to the adhesion results, the chicken isolate RO37 had the highest internalization levels compared to *C. jejuni* 81-176.

### Motility of poultry isolated *C. jejuni* strains

**Figure 2:**
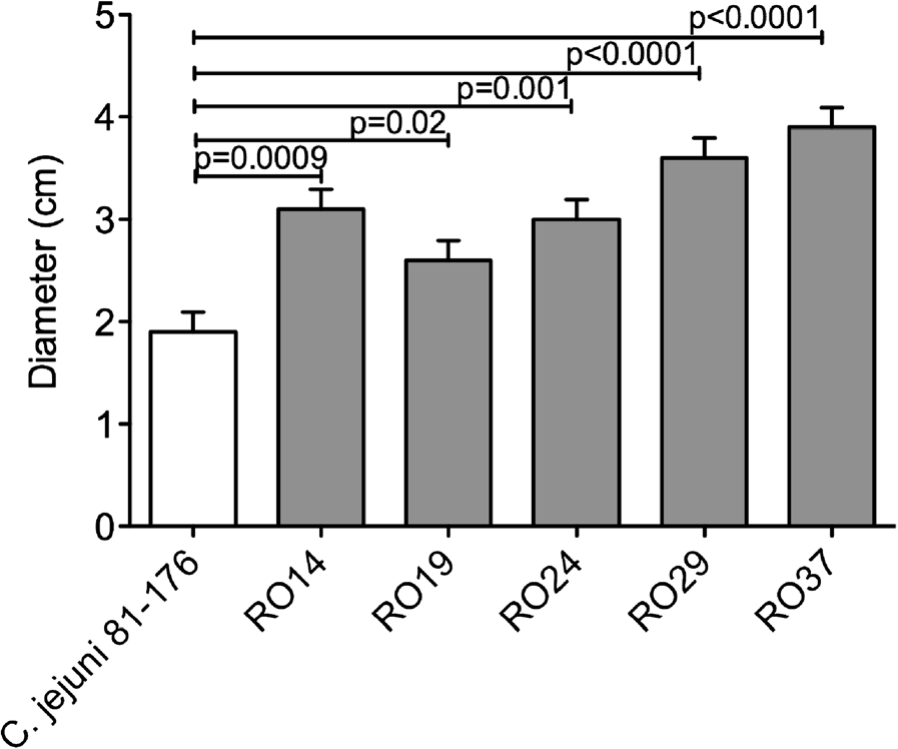
**Motility of *C. jejuni *chicken isolates in 0.4% motility agar.** Motility of *C. jejuni* chicken isolates RO14, RO19, RO24, RO29 and RO37 by comparison with the highly virulent strain *C. jejuni* 81-176. Size of the motility halo after 48 h at 37°C. Results are the mean of three separate experiments. Statistical significance (Student’s *t* test) relative to the level of *C. jejuni* 81-176 strain is indicated.

Motility was previously described as an important virulence factor in *C. jejuni* described [9]. Based on our results, showing improved adhesion and invasion of all the chicken isolates, next we have investigated their motility. As expected all these new strains showed increased motility compared to the control strain *C. jejuni* 81-176 as judged by the increased migration in semisolid agar (Figure 2), which correlates with their increase in virulence. Statistically all the increases in motility were significant but similarly as for adhesion/invasion the most significant increase was observed for strain RO37 (p < 0.0001).

### Resistance to human serum

**Figure 3:**
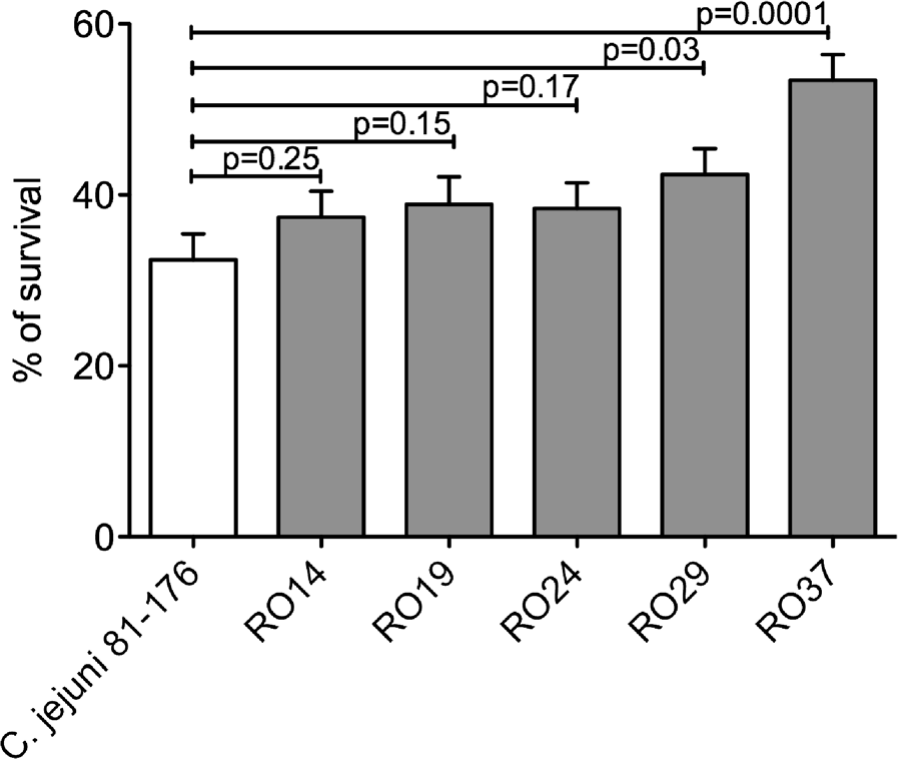
**Serum resistance.** Serum resistance of *C. jejuni* chicken isolates RO14, RO19, RO24, RO29 and RO37 by comparison to *C. jejuni* 81-176. The survival rate is defined as the number of *C. jejuni* colonies isolated following exposure to human serum divided by the number of colonies surviving in heat-inactivated serum, expressed as a percentage. The experiment was performed in triplicate. The error bars represent standard deviations. Statistical significance was assessed with Student’s *t* test.

The ability of *C. jejuni* to cause systemic infection in imuncompromised hosts it was suggested to be related to the increase in resistance to the bactericidal activity present in the serum of these patients [14]. Suerly, this will not be the case with the human serum (Invitrogen) and we have investigated the serum survival rate of these newly chicken isolated strains. As shown in Figure 3 the isolates RO14 (p = 0.25), RO19 (p = 0.15) and RO24 (p = 0.17) had a better rate of survival in human serum but not statistically significant. However, the isolates RO29 (p = 0.03) and RO37 (p = 0.0001) had a significantly incresed serum survival rate (Figure 3) compared to the control strain *C. jejuni* 81-176.

### Resistance to bile salts

**Figure 4:**
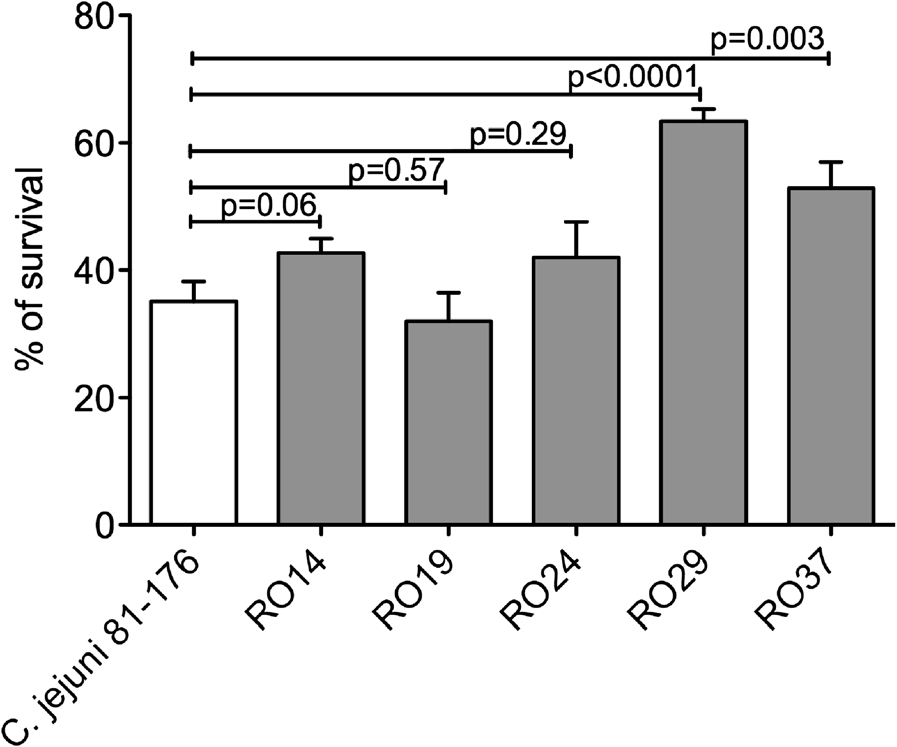
**Bile resistance.** The ability of *C. jejuni* chicken isolates (RO14, RO19, RO24, RO29, RO37) and *C. jejuni* 81-176 to survive to bile salts in Mueller Hinton medium was studied. Following agar growth bacteria were transferred into 50 mL of medium containing 0.5% bile salts. Samples were removed after 24 h and diluted prior plating. The surviving cells were counted after 42 h incubation. The experiments were done in triplicates and Student *t* test was used for statistical significance.

In order to fulfill its pathogenicity, *in vivo*, *C. jejuni* must resist the deleterious actions of bile in order to survive in the human gastrointestinal tract. This experiment aimed to observe any differences between the response of *C. jejuni* 81-176 and chickens isolates to elevated levels of bile salts in the media and, as a result, speculate on their behaviour within their normal habitat of the intestine. The chicken isolates RO14, RO19 and RO24 had a percentage of survival similar to *C. jejuni* 81-176 (Figure 4). The most significant increase in survival was observed for the chicken isolate RO29 where the increase was over 30% (p < 0.0001). The chickens isolate RO37, which has been proven also to be the most virulent (Figure 1B) an increase of in bile resistance of 15% was detected.

### Antimicrobial resistance

For each *C. jejuni* isolate antibiotic inhibition zones to the various antibiotics were recorded and compared to the resistance of *C. jejuni* 81-176 control strain. The antibiotic profiles of the *C. jejuni* poultry isolates were different to the profiles of *C. jejuni* 81-176 control strain which shwed restitace only to gentamicin (Table 1). Only two of the chicken isolates were senzitive to nalidixic acid (RO19 and RO24). The resistance to erythromycin and ampicillin was detected for all the chicken isolates but interestingly not for the control strain. For ciprofloxacin and amixicillin-clavulinic acid the resistance profiles of the chicken isolates matched the profile of *C. jejuni* 81-176, lack of resistance being detected for all of them. Resistance to gentamicin was detected for RO19, RO29 and RO37 chicken isolates and also for the control strain *C. jejuni* 81-176.

**Table 1:** **Antimicrobial resistance rates of *C. jejuni *** **isolated from poultry**

**Specie**	**Nalidixic acid**	**Ciprofloxacin**	**Erythromycin**	**Ampicillin**	**Amoxicilin-clavulanic acid**	**Gentamicin**
*C. jejuni* 81-176	-	-	-	-	-	+
RO14	+	-	+	+	-	-
RO19	-	-	+	+	-	+
RO24	-	-	+	+	-	-
RO29	+	-	+	+	-	+
RO37	+	-	+	+	-	+

## Discussions

In 2010 the European Centre for Disease Prevention and Control (EFSA) reported that campylobacteriosis is still the most common zoonosis in the European Union (EU) [15]. In the past ten years a total of 13 new countries in Eastern Europe joined the EU and currently very little is known in regards to the virulence of *C. jejuni* strains currently present in those areas. In humans the most common cause of campylobacter enteritis is caused by *C. jejuni* which commonly colonizes the intestinal tract of chickens [16]. It is now recognized that in some countries in Eastern Europe, especially in suburban areas, much of the population grow chicken broilers for their own consumption. Therefore our study focused on the isolation and investigation of the virulence characteristics of *C. jejuni* strains isolated from households in rural areas in the regions of Banat and Transylvania of Romania.

An essential feature in the development of an infectious disease is the initial transport of the bacterial pathogen to the host. In chickens, *C. jejuni* will only colonize the mucosa without entering the epithelial cells. The success in colonization will depend on the ability of bacteria to compete successfully with the host’s normal microbiota for essential nutrients. However, in the human gut *C. jejuni* will have to will have to not only to perform colonization but also internalization in order to cause infection. In order to achieve this the pathogen will have fulfill few conditions for an efficient infection that includes: high motility [17], to out rule the detrimental effects of the bile [18], to express efficiently adhesion molecules [19,20] and if the infection becomes systemic to survive the antimicrobial effect of the human serum [21].

The mechanisms by which *Campylobacter* species cause diarrhea and possible subsequent sequelae are not well defined. Invasion and adhesion are generally an important pathogenic factor and it was reported for clinical and non-clinical isolates [22,23]. In our experiments all the chicken isolates were significantly more adherent and invasive compared to the control strain *C. jejuni* 81-176. This finding is consistent with previous reported data showing that chicken isolates are more or at least as invasive as human isolates [11]. Studies with isolated primary cells from chickens showed that *C. jejuni* was able to invade chicken cells [24] and that invasion is largely dependent on strain isolates.

Motility plays and important role in *Campylobacter* colonization, since the organism has to penetrate mucus in order to adhere to and invade surface epithelial cells. In our study the motility of the five chicken isolates, found to have different virulence potential following examination on motility agar. The role of motility has been previously demonstrated in several animal models [25] and also on human volunteer experiments [26]. We have also shown previously that diminished motility results in reduced ability of *C. jejuni* 81-176 to infect human epithelial cells [13]. The increased motility of the new chicken isolates will be of great importance for bacterial host interactions during infection and will impact on their the ability to cause disease.

When systemic *C. jejuni* occurs in an immunocompromised individual the bacterium will have to overcome the antimicrobial activity of the human serum. This antibacterial activity of a serum component is an important innate defense against bacteria within the vascular compartment. Using fresly pooled human serum we show that only two of the chicken isolates (RO29 and RO37) were significantly more resistant compared to *C. jejuni* 81-176. The resistance to human serum, of these two strains, might arise from unique surface charcateristics allowing them to evade the bactericidal effect of serum complements. How and why these new isolates are more virulent requires investigation of important virulence factors including capsule polysaccharide (CPS). The amount of CPS was shown to be important for the survival of *C. jejuni* to human serum [27]. One important feature involved in serum resistance reffers to the protein structure of the S-layer which is also involved in mediating bacterial resistance to human serum. These proteins will confer resistance to complement-mediated killing by using opsonic antibodies directed against the S-layer [28].

Little is known on the bile tolerance of Gram-negative bacteria, but it is belived that they are more resistant to bile than Gram-positive bacteria. An adaptation that is vital to the survival of *Campylobacter* in the gut and, hence, its success in causing infection, is its ability to withstand the presence of bile salts in the surrounding environment. Such an adaptation will serve as a competitive advantage over other bacteria colonising the gut. The volume of the bile salt pool in man has been estimated as either 4 g or 7.6 mmol was identified as 7.6 ± 0.9 mmol, which is secreted and reabsorbed at least twice during the digestion of a single meal [29]. Our results show that, at least for two of the chicken isolates (RO29 and RO37) their survival rates in the presence of bile acids were significantly higher when compared to the survival rate of *C. jejuni* 81-176. This increase in survival to the bile acids could have a positive impact on the pathogenic abilities of this two strains and corelate to the other virulence traits here identified.

In most cases *Campylobacter* infections are acute and self-limited in nature and will not require antibiotic treatment, unless infections occur in immunocompromised patients. Unfortunately, not all the laboratories will routinely perform antimicrobial testing of new *C. jejuni* isolates. In our experiments the resistance to erythromycin was very high in all *C. jejuni* chicken isolates similarly to what was previously reported in the literature [30]. The resistance to erythromycin, in the new *C. jejuni* chicken isolates, was not acquired via plasmid conjugation since the erythromycin resistance is unrelated to the presence of plasmid DNA and therefore is considered to be chromosomally mediated [31]. The production of beta-lactamases in bacteria is associated with resistance to ampicillin and in our experiments all the *C. jejuni* chicken isolates are highly resistant to ampicillin.

However, when an antibiotic resistant to beta-lactamases is used (combination of amoxicillin and clavulanic acid), all the new *C. jejuni* chicken isolates were not able to survive.

In conclusion, we have shown that *C. jejuni* chicken isolates exhibit a range of invasion phenotypes with hyper invasiveness being more frequently observed among *C. jejuni* chicken isolates. However, extrapolating these results to the host it is premature at this stage because *C. jejuni* infection is highly dependent on the physiological and immunological condition of the host.

## Competing interests

The authors declare that they have no competing interests.

## Authors’ contributions

LS, NC and DD designed the study; AT, CJ performed experiments; NC wrote the paper. All authors read and approved the final manuscript.
